# Changing Predominance of Norovirus Recombinant Strains GII.2[P16] to GII.4[P16] and GII.4[P31] in Thailand, 2017 to 2018

**DOI:** 10.1128/spectrum.00448-22

**Published:** 2022-05-12

**Authors:** Pattara Khamrin, Kattareeya Kumthip, Arpaporn Yodmeeklin, Nutthawadee Jampanil, Phitchakorn Phengma, Panuwat Yamsakul, Shoko Okitsu, Takeshi Kobayashi, Hiroshi Ushijima, Niwat Maneekarn

**Affiliations:** a Department of Microbiology, Faculty of Medicine, Chiang Mai University, Chiang Mai, Thailand; b Center of Excellence in Emerging and Re-emerging Diarrheal Viruses, Chiang Mai University, Chiang Mai, Thailand; c Department of Food Animal Clinic, Faculty of Veterinary Medicine, Chiang Mai University, Chiang Mai, Thailand; d Division of Microbiology, Department of Pathology and Microbiology, Nihon University School of Medicine, Tokyo, Japan; e Department of Virology, Research Institute for Microbial Diseases, Osaka University, Osaka, Japan; Wright State University

**Keywords:** diarrhea, norovirus, recombination, Thailand

## Abstract

Human norovirus is a major virus that causes acute gastroenteritis in all age groups. Recently, norovirus recombinant strains have been reported as the cause of norovirus outbreaks. This study has investigated the distribution of norovirus genotypes and recombinant strains circulating in children hospitalized with diarrhea in Chiang Mai, Thailand from 2017 to 2018. A total of 882 stool specimens were tested for the presence of norovirus GI and GII by reverse transcription-PCR (RT-PCR) assay. Genotypes of the viruses were assessed by partial VP1 nucleotide sequencing and the representative strains were further characterized for norovirus recombinant strains by amplification of ORF1 (RdRp)/ORF2 (VP1 capsid) junction region. From a total of 882 stool samples, 131 (14.9%) were positive for norovirus, of which the majority of norovirus genogroups were norovirus GII, and only one was identified as norovirus GI. A wide variety of norovirus genotypes were detected in this study, including GI.5, GII.2, GII.3, GII.4, GII.6, GII.7, GII.13, GII.14, and GII.17 with the predominance of GII.2 (62.5%) in 2017 and GII.4 (57.0%) in 2018. Nevertheless, it should be noted that GII.4 remained the most predominant genotype (50.4%) in overall prevalence. Analysis of norovirus recombination revealed that several norovirus recombinant strains (GII.2[P16], GII.3[P16], GII.4[P16], GII.4[P31], GII.6[P7], GII.13[P16], and GII.14[P7]) had been identified with the predominance of GII.2[P16] in 2017 and changed to GII.4[P16] and GII.4[P31] in 2018. In conclusion, this study reported the detection of a wide variety of norovirus genotypes and several norovirus recombinant strains in Chiang Mai, Thailand from 2017 to 2018.

**IMPORTANCE** In the present study, the prevalence of norovirus infection in children with acute gastroenteritis in Chiang Mai, Thailand between 2017 and 2018 was 14.9%. A variety of norovirus genotypes were detected, including GI.5, GII.2, GII.3, GII.4, GII.6, GII.7, GII.13, GII.14, and GII.17 with the predominance of GII.4 genotype. In addition, several norovirus recombinant strains (GII.2[P16], GII.3[P16], GII.4[P16], GII.4[P31], GII.6[P7], GII.13[P16], and GII.14[P7]) had been identified. Our results revealed that GII.2[P16] was a predominant strain till the end of 2017 and then was replaced by GII.4[P16] and GII.4[P31] in 2018. The findings imply that norovirus recombinant strains emerged in Chiang Mai, Thailand and that circulating strains changes over time.

## INTRODUCTION

Acute gastroenteritis is one of the important causes of morbidity and mortality in infants and young children worldwide. Morbidity and mortality of diarrheal diseases in children under 5 years old in many countries are attributable to viral infection (1). Human norovirus is an important enteric pathogen that is associated with acute gastroenteritis (2). The virus in the genus *Norovirus*, family *Caliciviridae*, is a small non-enveloped with a positive-sense, single-stranded RNA genome of approximately 7.3 to 8.3 kb in length (3). The genome is organized into three open reading frames (ORF1 to ORF3). ORF1 encodes nonstructural proteins including RNA-dependent RNA polymerase (RdRp), whereas ORF2 and ORF3 encode the major and minor structural proteins VP1 and VP2, respectively (3, 4). Based on the diversity of the complete VP1 capsid protein, noroviruses are classified into at least 10 genogroups (GI to GX). Norovirus GI and GII are the most predominant genogroups associated with human diseases and are further subdivided into at least nine and 26 genotypes, respectively (5). The norovirus typing, based on nucleotide diversity of RdRp has also been proposed, and the dual typing systems of norovirus VP1 capsid type and RdRp type were recently updated (5–7). For the dual typing nomenclature, a capital P (for RdRp genotype) in the parenthesis is used following the VP1 capsid genotype such as GI.1[P1], GII.2[P16], etc. Based on nucleotide sequence diversity of the RdRp, noroviruses are divided into 60 P-genotypes (14 GI, 37 GII, 2 GIII, 1 GIV, 2 GV, 2 GVI, 1 GVII, and 1 GX), with two tentative P-genogroups and 14 tentative P-genotypes (5). Molecular epidemiological studies revealed that the norovirus GII.4 is responsible for most reported norovirus-associated outbreaks and sporadic cases for over 2 decades globally (8, 9). From the winter of 2014 to 2015, a novel GII.17 norovirus variant, named Kawasaki 2014, emerged and became the predominant genotype associated with acute gastroenteritis cases in Asia including Hong Kong (10), Japan (11), and China (12, 13). Recently, several studies of norovirus recombinant strains revealed that recombination often occurs near to or within the ORF1 (RdRp)/ORF2 (VP1) junction (14–17). The norovirus GII.2[P16] recombinant strain associated with acute gastroenteritis has emerged from 2016 to 2017 and spread in Europe (18, 19) and many countries in Asia, including China (20, 21), Hong Kong (22), Japan (23), Malaysia (24), Taiwan (25), and Thailand (26, 27). In Thailand, GII.2[P16] has been demonstrated not only as the cause of acute gastroenteritis but also detected in oysters that could be a potential source for foodborne transmission to humans (28). The outbreaks were also attributable to the emergence of GII.4[P16] and GII.4[P31] (29, 30). This study aimed to investigate the prevalence of norovirus and to identify the dynamics of norovirus recombinant strains circulating in children hospitalized with acute gastroenteritis in Chiang Mai, Thailand from 2017 to 2018.

## RESULTS

### Norovirus detection and genotype distribution.

**FIG 1:**
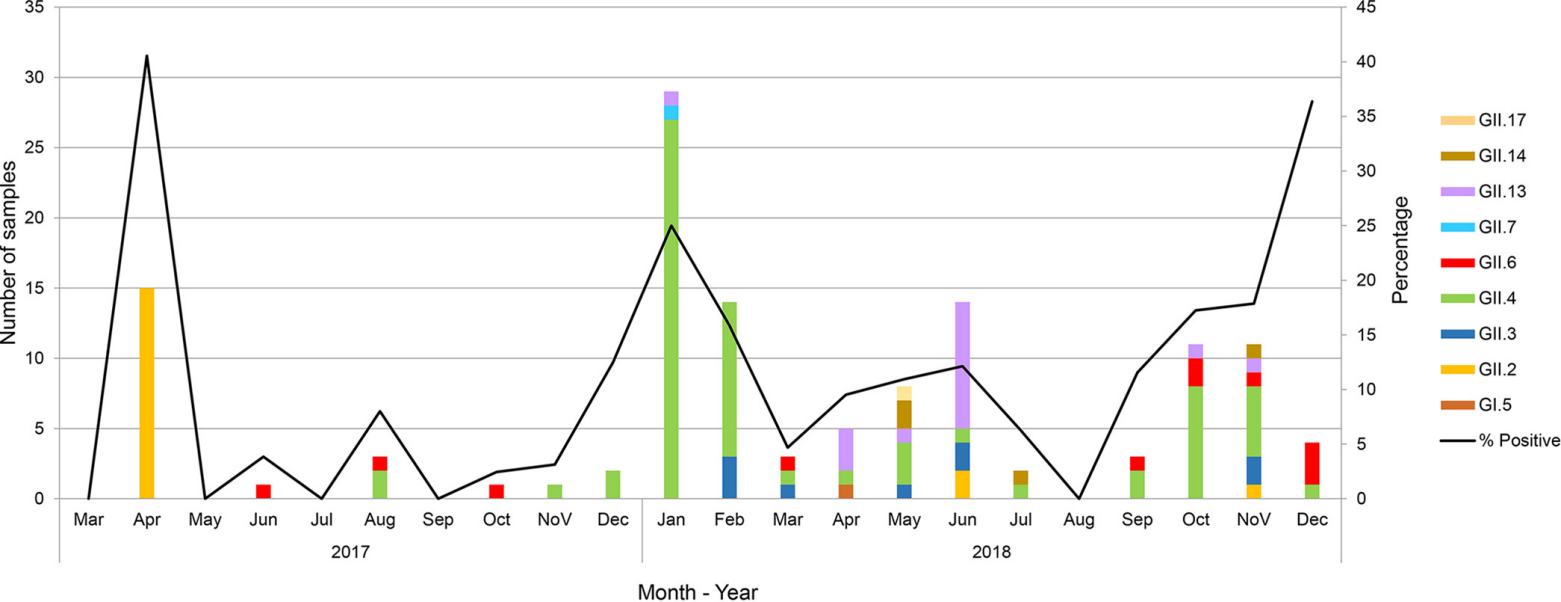
Seasonal distribution of norovirus detected in children with acute gastroenteritis from 2017 to 2018.

Among 882 stool samples tested, 14.9% (131 out of 882) were positive for norovirus as shown in Table 1. When the stool samples were stratified based on the year of collection, the prevalence of norovirus infection in 2017 was 9.8% (24 out of 246) and 16.8% (107 out of 636) in 2018. The majority of norovirus detected in this study (99.2%; 130 out of 131) was norovirus GII and only 0.8% (one out of 131) was norovirus GI. None of norovirus GIV was detected in this study. All of the detected noroviruses were further characterized for their VP1 capsid genotypes by nucleotide sequencing. Eight genotypes of norovirus GII were identified, including GII.2, GII.3, GII.4, GII.6, GII.7, GII.13, GII.14, and GII.17. The norovirus GII.4 was the most common genotype (50.4%) detected in this study, followed by GII.13 (16.0%), GII.2 (13.7%), GII.6 (8.4%), GII.3 (5.3%), GII.14 (3.8%), GII.7 (0.8%), and GII.17 (0.8%). The only norovirus GI detected in this study was GI.5 genotype. It was interesting to point out that in 2017, only three genotypes of norovirus GII (GII.2, GII.4, and GII.6) were detected and GII.2 was the most common genotype with a prevalence of 62.5%. Unexpectedly, in the following year 2018, the prevalence of GII.2 decreased abruptly down to only 2.8%. Nevertheless, the GII.4 which was detected at a lower prevalence (20.8%) in 2017 rose to 57.0% in 2018. The seasonality of norovirus infection in pediatric patients with acute gastroenteritis in Thailand is shown in Fig. 1. In general, norovirus infection was found throughout the year with high prevalence in dry and cool months (January to February, and October to December) in Thailand.

**TABLE 1:** Detection rates and genotype distribution of norovirus in pediatric patients with acute gastroenteritis in Chiang Mai, Thailand from 2017 to 2028

Yr	Total samples	No. of norovirus positive	% Norovirus positive	Norovirus genotypes (%)								
				GI.5	GII.2	GII.3	GII.4	GII.6	GII.7	GII.13	GII.14	GII.17
2017	246	24	9.8	0	15 (62.5)	0	5 (20.8)	4 (16.7)	0	0	0	0
2018	636	107	16.8	1 (0.9)	3 (2.8)	7 (6.6)	61 (57.0)	7 (6.6)	1 (0.9)	21 (19.6)	5 (4.7)	1 (0.9)
Total	882	131	14.9	1 (0.8)	18 (13.7)	7 (5.3)	66 (50.4)	11 (8.4)	1 (0.8)	21 (16.0)	5 (3.8)	1(0.8)

### Phylogenetic analysis of norovirus VP1capsid gene.

**FIG 2:**
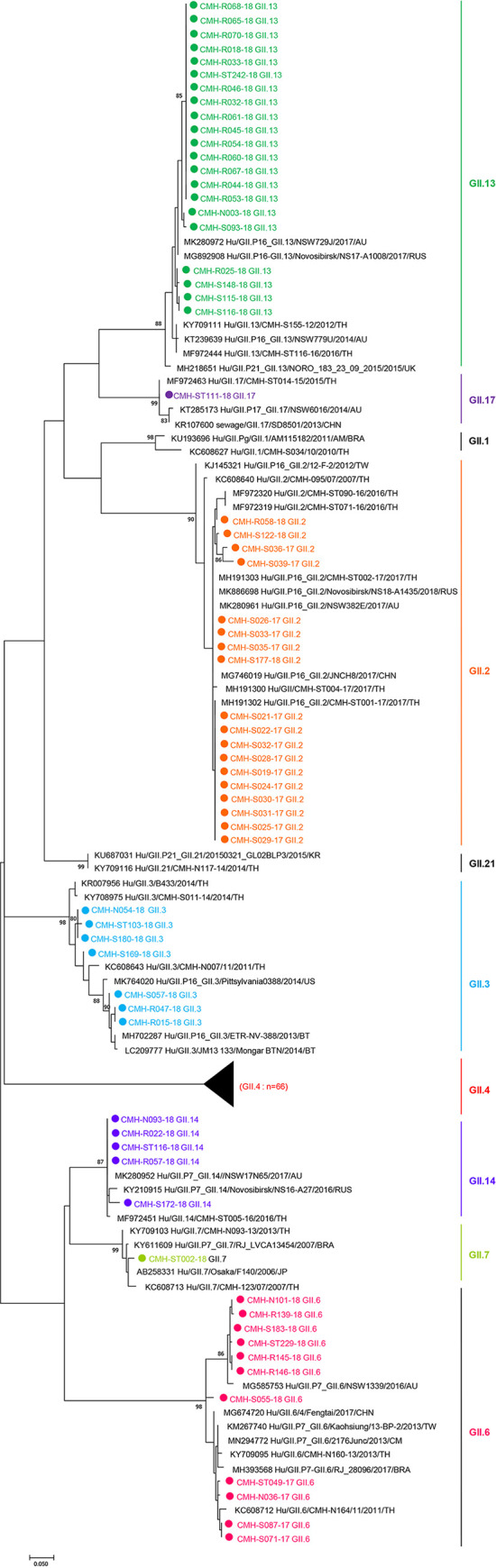
Phylogenetic analysis of the partial VP1 nucleotide sequences (295 nt) of norovirus strains detected in this study. The scale bar indicates nucleotide substitutions per site and bootstrap values are indicated for the corresponding nodes. The norovirus GII strains detected in this study are indicated in solid circle.

The phylogenetic tree of the partial VP1 nucleotide sequences (295 nt) of norovirus GII detected in this study was constructed (Fig. 2). The majority of norovirus detected in this study belonged to norovirus GII.4 (*n* = 66). All 66 strains of norovirus GII.4 were GII.4 Sydney variants that showed a close genetic relationship with other GII.4 Sydney variant strains reported previously worldwide with nucleotide sequence identities ranging from 80.7% to 96.9%. The nucleotide sequence identities among 66 GII.4 Sydney strains detected in this study ranging from 91.5% to 100%. The norovirus GII.13 strains, the second most common genotype detected in this study (*n* = 21), formed a cluster with norovirus GII.13 reference strains with nucleotide sequence identities ranging from 80.7 to 95.1% and showed close similarity sequences with GII.13 strains previously detected in 2017 from Australia and Russia. For norovirus GII.2 (*n* = 18), all of the strains detected in this study were located in the same branch and were closely related to norovirus GII.2 reference strains reported previously in 2016 and 2017 from Thailand and other GII.2 reference strains from Russia, Australia, and China with the nucleotide sequence identities ranging from 77.8 to 99.6%. Phylogenetic analysis of norovirus GII.6 (*n* = 11) strains demonstrated that they were closely related to GII.6 reference strains reported previously from Thailand and many countries worldwide including China, Taiwan, Australia, Brazil, and Cameroon with the nucleotide sequence identities ranging from 77.3 to 98.9%. It was observed that norovirus GII.6 strains detected in this study were phylogenetically separated into two clusters based on the year of specimen collection. The strains detected in 2017 were most closely related to the Thai norovirus GII.6 strain detected in Chiang Mai in 2011, while the strains detected in 2018 were clustered together with the norovirus GII.6 Australian strain isolated in 2016. Seven strains of norovirus GII.3 were most closely related to the norovirus GII.3 genotype reported previously from Thailand in 2011 and 2014, Bhutan in 2013 and 2014, and Australia in 2011, with the nucleotide sequence identities ranging from 71.8% to 98.6%. For norovirus GII.14, all five GII.14 strains detected in this study were located closely together in the same branch with norovirus GII.14 reference strains reported previously in 2016 and 2017 from Thailand, Australia, and Russia with the nucleotide sequence identities ranging from 79.6% to 97.8%. One norovirus GII.7 showed a close genetic relationship with GII.7 reference strains reported previously from Thailand, Japan, and Brazil with the nucleotide sequence identities ranging from 76.8% to 97.5%. In addition, one norovirus GII.17 detected in this study shared nucleotide sequence identities ranging from 78.%6 to 98.2% with norovirus GII.17 reference strains reported from Thailand, China, and Australia.

### Recombination analysis of norovirus GII.

**FIG 3:**
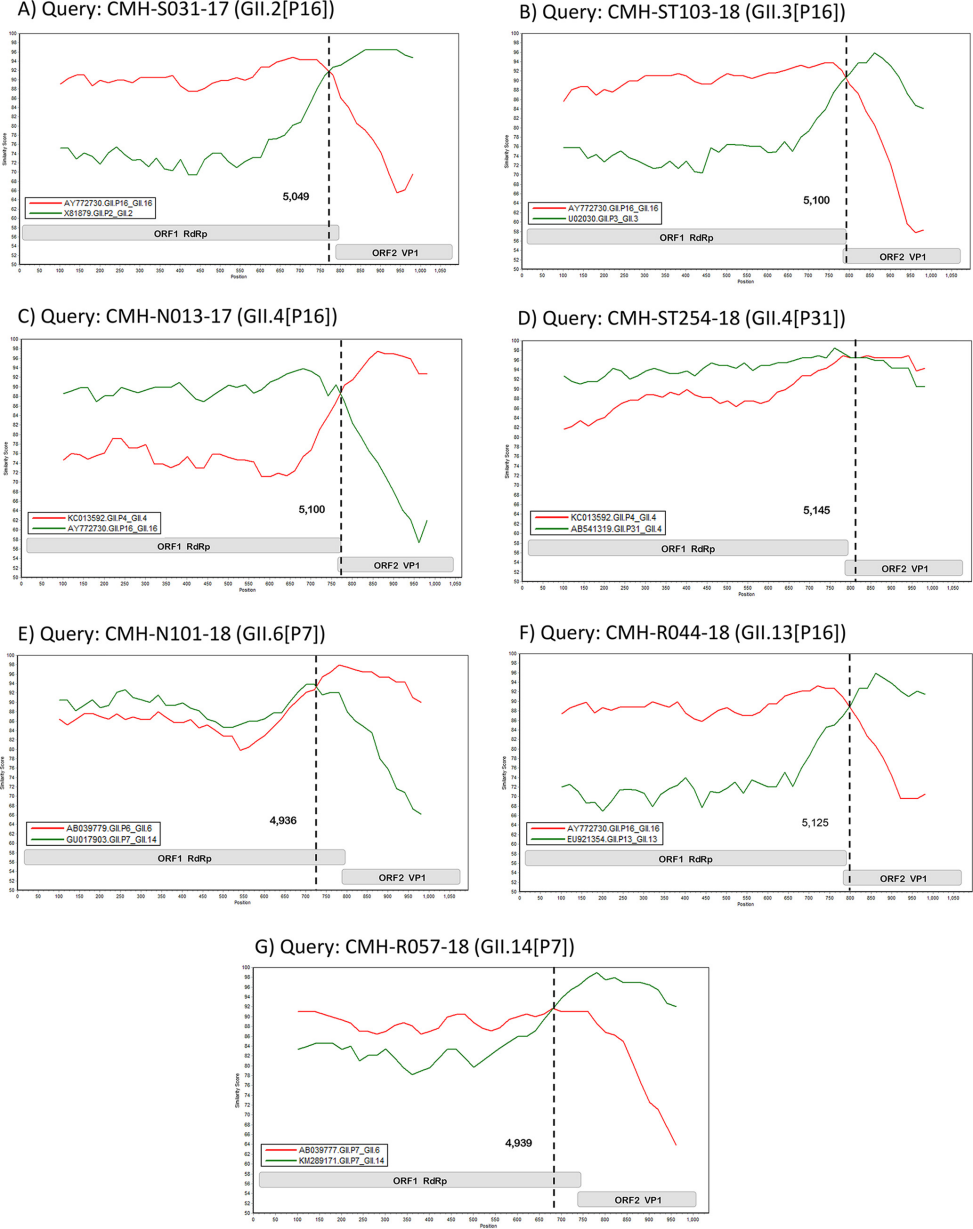
Similarity plots for the seven norovirus recombination patterns detected in Chiang Mai, Thailand from 2017 to 2018. The vertical axis indicates nucleotide sequence identity (%) between sequences of norovirus from this study and the reference strains. The vertical dashed line defines the predicted recombination site.

Of 130 norovirus GII positive samples, 43 representative strains were selected for further analysis for norovirus recombination. Sequence analysis revealed that 41 out of 43 (95.3%) norovirus GII were found to be recombinant strains. The ORF1/ORF2 junction sequences were analyzed by the SimPlot software and confirmed the recombination breakpoint by the RDP4 program. Seven norovirus recombination patterns including GII.2[P16], GII.3[P16], GII.4[P16], GII.4[P31], GII.6[P7], GII.13[P16], and GII.14[P7] were identified (Table 2). Twelve strains of GII.2[P16] recombinant strains were detected both in 2017 and 2018, and the recombination breakpoints of these strains were located at nucleotide positions of 5,029, 5,039, and 5,049 within the 3′-end region of ORF1(Table 2). The similarity plot of the representative GII.2[P16] recombinant strains (CMH-S031-17) is shown in Fig. 3A. Two GII.3[P16] recombinant strains were detected only in 2018, and the recombination breakpoints located at the nucleotide positions 5,100 within the overlapping region of ORF1/2 and 5,130 of the ORF2 region. The similarity plot of the representative GII.3[P16] recombinant strains (CMH-ST103-18) is shown in Fig. 3B. For GII.4[P16] recombinant strains, the majority of these strains were detected in 2018, while only one was detected in 2017. The recombination breakpoints of these recombinant strains located at different nucleotide positions ranging from 5,049 to 5,100 within the 3′-end region of ORF1 and overlapping region of ORF1/ORF2 (Table 2). The similarity plot of the representative GII.4[P16] recombinant strains (CMH-N013-17) is shown in Fig. 3C. Among six GII.4[P31] recombinant strains, five were detected in 2018 and only one was detected in 2017, the recombination breakpoints of these recombinant strains were located at different nucleotide positions ranging from 5,105 to 5,165 within the 5′-end region of ORF2 (Table 2). The similarity plot of the representative GII.4[P31] strains (CMH-ST254-18) is shown in Fig. 3D. Two noroviruses GII.6[P7] recombinant strains were detected both in 2017 and 2018. The recombination breakpoints of these strains are located at nucleotide positions 4,959 and 4,936, respectively, within the 3′-end region of ORF1(Table 2). The similarity plot of the representative GII.6[P7] recombinant strains (CMH-N101-18) is shown in Fig. 3E. For GII.13[P16] recombinant strains, all of these seven recombinant strains were detected in 2018, and the recombination breakpoints located at nucleotide positions of 5,110, 5,122, and 5,125, within the 5′-end region of ORF2 (Table 2). The similarity plot of the representative GII.13[P16] recombinant strains (CMH-R044-18) is shown in Fig. 3F. For the GII.14[P7] recombinant strains, all of them were detected only in 2018, and the recombination breakpoints were located at nucleotide positions 4,939 and 4,949 within the 3′-end region of ORF1 (Table 2). The similarity plot of the representative GII.14[P7] recombinant strains (CMH-R057-18) is shown in Fig. 3G. The significant recombinant events of all these strains were detected at the *P-*values ranging from 9.2 × 10^−14^ to 1.7 × 10^−2^ (Table 2). Analysis of norovirus GII recombinant strains detected in this study revealed that GII.2[P16] was the majority of norovirus recombinant strains circulating in Chiang Mai, Thailand in 2017, and then in 2018, it was replaced by norovirus GII.4[P16] and GII.4[P31] recombinant strains.

**TABLE 2:** Analysis of norovirus recombinant strains in pediatric patients with acute gastroenteritis in Chiang Mai, Thailand from 2017 to 2018

Sample code	Norovirus genotypes		Reference strains used for Simplot analysis (accession no.)		Predicted recombination nt positions	Breakpoint on ORF	P-value (Bootscan)
	ORF1 (RdRp)	ORF2 (VP1)	RdRp genotype	VP1 genotype			
CMH-S087-17	GII.P7	GII.6	GII.14[P7] (GU017903)	GII.6[P6] (AB039779)	4959	ORF1	4.9 × 10^−6^
CMH-N101-18	GII.P7	GII.6			4936	ORF1	1.8 × 10^−3^
CMH-ST002-18	GII.P7	GII.7	GII.7[P7] (KJ196295)	GII.7[P7] (AF414409)	No evidence of recombination		
CMH-S172-18	GII.P7	GII.14	GII.6[P7] (AB039777)	GII.14[P7] (KM289171)	4949	ORF1	1.1 × 10^−10^
CMH-ST116-18	GII.P7	GII.14			4949	ORF1	3.4 × 10^−8^
CMH-R057-18	GII.P7	GII.14			4939	ORF1	1.3 × 10^−6^
CMH-S093-18	GII.P16	GII.13	GII.16[P16] (AY772730)	GII.13[P13] (EU921354)	5110	ORF2	1.1 × 10^−10^
CMH-S115-18	GII.P16	GII.13			5125	ORF2	9.2 × 10^−5^
CMH-S116-18	GII.P16	GII.13			5125	ORF2	9.2 × 10^−5^
CMH-R025-18	GII.P16	GII.13			5122	ORF2	5.4 × 10^−11^
CMH-R044-18	GII.P16	GII.13			5125	ORF2	7.2 × 10^−11^
CMH-R053-18	GII.P16	GII.13			5125	ORF2	1.3 × 10^−9^
CMH-R060-18	GII.P16	GII.13			5125	ORF2	5.7 × 10^−10^
CMH-S021-17	GII.P16	GII.2	GII.16[P16] (AY772730)	GII.2[P2] (X81879)	5049	ORF1	8.7 × 10^−12^
CMH-S022-17	GII.P16	GII.2			5049	ORF1	3.9 × 10^−12^
CMH-S025-17	GII.P16	GII.2			5049	ORF1	3.0 × 10^−11^
CMH-S026-17	GII.P16	GII.2			5029	ORF1	3.2 × 10^−12^
CMH-S029-17	GII.P16	GII.2			5049	ORF1	3.0 × 10^−11^
CMH-S030-17	GII.P16	GII.2			5049	ORF1	7.8 × 10^−11^
CMH-S031-17	GII.P16	GII.2			5049	ORF1	3.0 × 10^−11^
CMH-S033-17	GII.P16	GII.2			5039	ORF1	7.4 × 10^−12^
CMH-S035-17	GII.P16	GII.2			5039	ORF1	5.8 × 10^−13^
CMH-S122-18	GII.P16	GII.2			5039	ORF1	8.4 × 10^−11^
CMH-S177-18	GII.P16	GII.2			5039	ORF1	1.4 × 10^−12^
CMH-R058-18	GII.P16	GII.2			5039	ORF1	9.9 × 10^−13^
CMH-S057-18	GII.P16	GII.3	GII.16[P16] (AY772730)	GII.3[P3] (U02030)	5130	ORF2	8.7 × 10^−10^
CMH-ST103-18	GII.P16	GII.3			5100	ORF1/2	1.5 × 10^−9^
CMH-N013-17	GII.P16	GII.4	GII.16[P16] (AY772730)	GII.4[P4] (KC013592)	5100	ORF1/2	9.2 × 10^−14^
CMH-S079-18	GII.P16	GII.4			5090	ORF1/2	1.3 × 10^−10^
CMH-N002-18	GII.P16	GII.4			5049	ORF1	1.3 × 10^−13^
CMH-N010-18	GII.P16	GII.4			5090	ORF1/2	6.7 × 10^−11^
CMH-N016-18	GII.P16	GII.4			5090	ORF1/2	6.6 × 10^−11^
CMH-N020-18	GII.P16	GII.4			5100	ORF1/2	1.1 × 10^−11^
CMH-N033-18	GII.P16	GII.4			5090	ORF1/2	7.5 × 10^−11^
CMH-N039-18	GII.P16	GII.4			5069	ORF1	9.2 × 10^−14^
CMH-ST095-18	GII.P16	GII.4			5100	ORF1/2	3.8 × 10^−11^
CMH-ST111-18	GII.P17	GII.17	GII.17[P17] (AB983218)	GII.17[P17] (LC043167)	No evidence of recombination		
CMH-S118-17	GII.P31	GII.4	GII.4[P31] (AB541319)	GII.4[P4] (KC013592)	5165	ORF2	3.5 × 10^−2^
CMH-S062-18	GII.P31	GII.4			5145	ORF2	3.8 × 10^−2^
CMH-ST023-18	GII.P31	GII.4			5105	ORF2	1.7 × 10^−2^
CMH-ST254-18	GII.P31	GII.4			5145	ORF2	5.8 × 10^−3^
CMH-ST258-18	GII.P31	GII.4			5145	ORF2	2.1 × 10^−3^
CMH-R144-18	GII.P31	GII.4			5119	ORF2	2.2 × 10^−3^

## DISCUSSION

In Thailand, norovirus infections in patients with acute gastroenteritis reported from 2000 to 2016 from several regions of Thailand revealed the overall prevalence of norovirus infection at 15.1% and the infection was more common in infants and young children than in adults (31). In the present study, the prevalence of norovirus GI and GII infection in children with acute gastroenteritis in Chiang Mai, Thailand between 2017 and 2018 was 14.9%, which is more or less the same as those of the whole country (31). For norovirus GIV, although the norovirus GIV was detected in environmental water in Chiang Mai, Thailand in 2016 and 2017 (32), unfortunately, none of norovirus GIV was detected in this set of stool samples collected from children with diarrhea. The potential sources of norovirus GIV spreading to the human population are yet inconclusive. The burden of norovirus GIV in humans requires further investigation.

The continued surveillance study of norovirus in children with acute gastroenteritis in Chiang Mai has been conducted since 2000 (33) and a wide variety of norovirus genotypes have been reported, including GI.3 to GI.9, GI.13, GI.14, GII.1 to GII.4, GII.6 to GII.10, GII.12 to GII.17, GII.20, and GII.21 (26, 31). It should be noted that GI.5, GII.2 to GII.4, GII.6, GII.7, GII.13, GII.14, and GII.17 genotypes remain currently circulating in pediatric patients with acute gastroenteritis in Chiang Mai, Thailand during 2017 and 2018.

Recombination is one of the mechanisms for the evolution of norovirus. The norovirus recombinant strains in pediatric patients with acute gastroenteritis in Chiang Mai, Thailand have been serially investigated from 2005 to 2015 (16). During these periods, a wide variety of norovirus recombinant strains, GII.P33 formerly Pg to GII.1, GII.P16 to GII.2, GII.P21 to GII.3, GII.P12 to GII.4, GII.P31 (formerly Pe) to GII.4, GII.P7 to GII.6, GII.P33 to GII.12, GII.P16 to GII.13, and GII.P7 to GII.14 have been reported. The GII.P16 to GII.2 recombinant strains were detected for the first time in Chiang Mai, Thailand in December 2013, whereas the GII.P31 to GII.4 strain was detected in 2014 and continued existing throughout 2015 (16). Nevertheless, the GII.P16-GII.4 recombinant strain has never been detected previously in Thailand and is reported for the first time in the present study. Several studies demonstrated that the RdRp GII.P16 has been demonstrated to associate with multiple capsid genotypes, including GII.1 to GII.4, GII.10, GII.12, and GII.13 (34, 35). In the present study, GII.P16 has been demonstrated to associate with GII.2, GII.3, GII.4, and GII.13. In fact, GII.P16 in association with GII.2 and GII.13 has been detected in Chiang Mai, Thailand from 2012 to 2013 (16). In addition, the emergence of norovirus GII.2[P16] recombinant strains was reported from 2016 to 2017 in other countries around the world, including Japan, China, Hong Kong, Taiwan, Malaysia, Germany, and France (18–25). In Thailand, norovirus GII.2[P16] was detected for the first time in Chiang Mai in 2013 (16) and re-emerged in January 2016, and become the predominant genotype in 2016 and 2017 (26). In the same period, GII.2[P16] was also reported as the cause of an outbreak associated with acute gastroenteritis in Bangkok, the capital city of Thailand (27). The present study, follow-up surveillance from 2017 to 2018 revealed that GII.2[P16] continued to be a predominant strain till the end of 2017 and then was replaced by GII.4[P16] and GII.4[P31] in 2018. The predominance of GII.4[P16] and GII.4[P31] over GII.2[P16] in 2018 corresponds directly to an upsurge of GII.4 and a sudden decrease of GII.2 from 62.5% in 2017 to 2.8% in 2018. The data implies that GII.4[P16] and GII.4[P31] are concurrently circulated in 2018 in children with acute gastroenteritis in Chiang Mai, a city located in the northern part of Thailand. The predominance of GII.4[P16] and GII.4[P31] have also been reported during the same period (2017 to 2018) in Bangkok, Thailand (30). In addition, it should be noted in this study that the RdRp GII.[P16] genotype is associated not only with GII.2 and GII.4 but also with GII.3 and GII.13 genotypes. It is interesting to point out that novel norovirus recombinant strain, GII.4[P16], emerged in 2015 in the United States (36), Since then it has been reported in sporadic cases and outbreaks of acute gastroenteritis in many countries around the world (30, 37). Additionally, the GII.4[P16] has been detected in environmental water in Korea (38). These accumulated reports of GII.4[P16] around the world indicated that this recombinant strain adapted well for maintenance in the human population. In conclusion, we have demonstrated the diversity of norovirus genotypes as well as recombinant strains circulating in pediatric patients hospitalized with acute gastroenteritis in Thailand in 2017 and 2018. The data provide useful information on the molecular epidemiology of norovirus and raise concerns about vaccine development for norovirus recombinant strains.

## MATERIALS AND METHODS

### Specimen collection.

Surveillance of norovirus was carried out from March 2017 to December 2018 in children younger than 5 years old who were hospitalized with acute gastroenteritis in Chiang Mai, Thailand. A total of 882 stool samples were collected from children admitted to five provincial/sub-provincial hospitals. The inclusion criteria of acute gastroenteritis were sudden passages of loose or watery stools for more than three times per day with the exclusion of bloody stools. All stool samples were stored at −20°C until use. The study was conducted with the approval of the Institutional Research Ethics Committee of the Faculty of Medicine, Chiang Mai University (MIC-2557-02710), and written informed consent was obtained from the participants’ guardians.

### RNA extraction and reverse transcription-PCR (RT-PCR).

The viral RNA genome was extracted from the supernatant of 10% fecal suspension in phosphate‐buffered saline (PBS) pH 7.4 using a Geneaid Viral Nucleic Acid Extraction Kit II (Geneaid, Taipei, Taiwan) according to the manufacturer’s protocol. Reverse transcription was performed using a RevertAid First Strand cDNA Synthesis Kit and random primer (Thermo Fischer Scientific, Waltham, MA, USA). The presence of noroviruses GI and GII were detected by PCR as described previously (36, 39, 40). For norovirus GI detection, the primers G1SKF and G1SKR were used to generate a PCR product size of 330 bp. For norovirus GII detection, primers COG2F and G2SKR were used to generate a PCR product size of 387 bp. A semi-nested PCR assay for norovirus GII was then performed using the primers G2SKF and G2SKR, which generated a PCR product size of 345 bp. In addition, the presence of norovirus GIV was determined by real-time PCR as described previously (41). All the detected norovirus strains were further characterized by nucleotide sequencing and phylogenetic analysis to determine their VP1 capsid genotypes.

### Norovirus recombination analysis.

To determine the potential recombination event of norovirus strains detected in this study, the partial ORF1 (RdRp)/ORF2 (VP1 capsid) junction region of norovirus strains obtained from norovirus-positive samples was amplified by semi-nested PCR. Briefly, the specific primers JV12Y and G2SKR were used for the first amplification to generate a PCR product size of 1,102 bp, and then semi-nested PCR was performed by using primers P290 and G2SKR to generate a PCR product size of 1,095 bp as described previously (42, 43). The recombination analysis was performed using the SimPlot software v.3.5.1 (44). The confidence interval calculation for the recombination breakpoint between the query strain and parental reference strains was determined by the Recombination Detection Program v.4.97 (RDP4) (45).

### Nucleotide sequence and phylogenetic analyses.

The amplified PCR products were purified using the Gel/PCR DNA Fragments Extraction Kit (Geneaid, Taipei, Taiwan) and subsequently sequenced (First BASE Laboratories Sdn Bhd Selangor Darul Ehsan, Malaysia). The nucleotide sequences obtained were analyzed using the ClustalX and the BioEdit software programs and then compared with those of other representative reference strains available in the GenBank database using the Basic Local Alignment Search Tool server (http://blast.ncbi.nlm.nih.gov/Blast.cgi). The VP1 capsid genotypes of noroviruses GI and GII were determined by using the Human Calicivirus Typing Tool (https://norovirus.ng.philab.cdc.gov/bctyping.html) (5, 7). The phylogenetic trees were constructed using MEGA7.0.26 software (46). The evolutionary history was generalized using the maximum likelihood method. The Kimura 2-parameter method was used as the best-fit evolutionary model and statistical analysis was performed using the bootstrap resampling method with 1,000 replicates.

### Data availability.

The nucleotide sequences of norovirus recombinant strains described in this study have been deposited in the GenBank database under the accession numbers: OK598006 to OK598048.
